# Adenoviral vector-mediated expression of a gene encoding secreted, EpCAM-targeted carboxylesterase-2 sensitises colon cancer spheroids to CPT-11

**DOI:** 10.1038/sj.bjc.6602362

**Published:** 2005-03-01

**Authors:** D Oosterhoff, R M Overmeer, M de Graaf, I H van der Meulen, G Giaccone, V W van Beusechem, H J Haisma, H M Pinedo, W R Gerritsen

**Affiliations:** 1 Department of Medical Oncology, Division of Gene Therapy, VU University Medical Center, PO Box 7057, 1007 MB Amsterdam, The Netherlands; 2 Department of Therapeutic Gene Modulation, University Center for Pharmacy, PO Box 196, 9700 AD Groningen, The Netherlands

**Keywords:** adenovirus, carboxylesterase, CPT-11, EpCAM, spheroid

## Abstract

CPT-11 (irinotecan or 7-ethyl-10[4-(1-piperidino)-1-piperidino] carbonyloxycamptothecin) is an anticancer agent in use for the treatment of colon cancer. In order to be fully active, CPT-11 needs to be converted into SN-38 (7-ethyl-10-hydroxycamptothecin) by the enzyme carboxylesterase (CE). In humans, only a minority of CPT-11 is converted to SN-38. To increase the antitumour effect of CPT-11 by gene-directed enzyme prodrug therapy, we constructed a replication-deficient adenoviral vector Ad.C28-sCE2 containing a fusion gene encoding a secreted form of human liver CE2 targeted to the surface antigen epithelial cell adhesion molecule (EpCAM) that is highly expressed on most colon carcinoma cells. By targeting CE2 to EpCAM, the enzyme should accumulate specifically in tumours and leakage into the circulation should be minimised. Ad.C28-sCE2-transduced colon carcinoma cells expressed and secreted active CE that bound specifically to EpCAM-expressing cells. In sections of three-dimensional colon carcinoma spheroids transduced with Ad.C28-sCE2, it was shown that C28-sCE2 was capable of binding untransduced cells. Most importantly, treatment of these spheroids with nontoxic concentrations of CPT-11 resulted in growth inhibition comparable to treatment with SN-38. Therefore, Ad.C28-sCE2 holds promise in gene therapy approaches for the treatment of colon carcinoma.

Conventional chemotherapy is not specific for tumour cells and therefore its administration is limited by side effects. These side effects might potentially be overcome by targeting chemotherapy specifically to tumour cells by gene-directed enzyme prodrug therapy (GDEPT). In GDEPT, a gene encoding a prodrug-converting enzyme is delivered to the tumour by, for example, an adenoviral vector. If the prodrug is administered it will be specifically converted to the active drug at the site of the tumour. This should increase the efficacy and decrease the side effects of chemotherapy. CPT-11 (irinotecan or 7-ethyl-10[4-(1-piperidino)-1-piperidino] carbonyloxycamptothecin) is an anticancer agent that is approved for first-line treatment of metastatic colon cancer. In order to be fully active, CPT-11 needs to be activated into the active compound SN-38 (7-ethyl-10-hydroxycamptothecin) by carboxylesterase (CE) enzymes (Tsuji *et al*, 1991; Satoh *et al*, 1994). Although SN-38 is detected in the plasma of cancer patients only minutes after administration of CPT-11 (Gupta *et al*, 1997), 90–95% of the prodrug is not converted to SN-38 (Rivory *et al*, 1997). A way to improve the antitumour effect of CPT-11 may be to use CPT-11 and CE in a GDEPT approach. Adenoviral-mediated expression of rabbit CE showed to sensitise efficiently a panel of tumour cell lines to CPT-11 (Wierdl *et al*, 2001). A human enzyme, however, has the advantage over a non-human enzyme that it will not lead to an immune response against the enzyme and subsequent enzyme inactivation. Kojima *et al* (1998) described the construction of a replication-deficient adenoviral vector containing the cDNA encoding human liver CE isoform 1 (CE1) (Kojima *et al*, 1998). Cell lines transduced with this virus and treated with CPT-11, however, showed only minimal antitumour effects. The liver CE isoform 2 (CE2) has a higher affinity and a higher conversion velocity of CPT-11 compared to CE1 (Humerickhouse *et al*, 2000). Therefore, we envisaged that human liver CE2 would be the best candidate to employ in a GDEPT approach to treat human colon cancer. Given the fact that current gene transfer technologies do not allow transduction of all tumour cells, a bystander effect is warranted to achieve effective kill of untransduced tumour cells. To improve the bystander effect of adenoviral vector-mediated GDEPT approaches, secreted and surface-tethered prodrug-converting enzymes have been investigated (Spooner *et al*, 2000; Weyel *et al*, 2000; Heine *et al*, 2001; Oosterhoff *et al*, 2003). We envisioned that a targeted, secreted form of CE2, consisting of the secreted form of CE2 (sCE2) fused to a tumour-specific scFv antibody would provide an enlarged bystander effect and would furthermore theoretically prevent leakage of the protein into the circulation, thereby reducing systemic side effects. Previously, we constructed a fusion protein in which sCE2 was fused to the human scFv antibody C28, which is directed to the tumour antigen Epithelial Cell Adhesion Molecule (EpCAM) (Oosterhoff *et al*, 2002). This fusion protein has potential utility for GDEPT of colon cancer, because EpCAM is highly overexpressed in colon cancer cells including distant metastases (Litvinov *et al*, 1994). Here, we describe the construction of a replication-deficient adenoviral vector containing the cDNA encoding the fully human fusion protein C28-sCE2. In a three-dimensional tumour spheroid model *in vitro*, we could demonstrate that the secreted fusion protein bound nontransduced cells and caused efficient killing of colon cancer cells in the presence of CPT-11.

## MATERIALS AND METHODS

### Cells and culture conditions

The colon cancer cell lines SW1398 and Colo205 and the ovarian cancer cell line A2780 (all cell lines were kindly provided by Dr E Boven, VUMC, Amsterdam, The Netherlands) were maintained in Dulbecco's modified Eagle's medium (DMEM) supplemented with 10% foetal calf serum (FCS), 50 IU ml^−1^ penicillin and 50 *μ*g ml^−1^ streptomycin (Invitrogen, Breda, The Netherlands), at 37°C in a 5% CO_2_ humidified atmosphere. The 293 cell line (ATCC, Manassas, VA, USA) was maintained in DMEM supplemented with 10% FCS, 50 IU ml^−1^ penicillin, 50 *μ*g ml^−1^ streptomycin and 2 mM L-glutamine (Invitrogen).

### Formation of colon cancer spheroids

In all 96-well plates (Greiner, Alphen aan den Rijn, The Netherlands) were coated with 2% agarose (Roche, Almere, The Netherlands) in PBS. Colon cancer SW1398 or Colo205 cells were plated (1 × 10^4^ cells well^−1^) and rotated overnight at 140 r.p.m. in a Heidolph Unimax incubator. By plating equal amounts of cells in each well and rotating them overnight, spheroids of similar sizes are formed. This allows direct comparison of different treatment modalities. After rotation, the formed spheroids were grown in a 5% CO_2_ humidified atmosphere at 37°C for 3 days before use in transduction experiments.

### Construction of Ad.C28-sCE2

The adenoviral vector Ad.C28-sCE2 was constructed using the AdEasy System (He *et al*, 1998). The plasmid pSTCF-C28-sCE2, containing the secreted, EpCAM-targeted CE2 (C28-sCE2) open reading frame (Oosterhoff *et al*, 2002) with a myc-6His tag at the C-terminus, was digested with *Pme*I and *Nhe*I, and the C28-sCE2 open reading frame was ligated into the *Xba*I- and *Eco*RV-linearised transfer vector pAdTrack-CMV. This construct contains a gene encoding green fluorescent protein (GFP) under the CMV promoter. Subsequently, the plasmid was digested with *Pme*I and cotransformed into *Escherichia coli BJ5183* cells with adenoviral backbone plasmid pAdEasy-1 to construct pAdEasy-C28-sCE2. After linearisation of this recombinant vector with *Pac*I, the plasmid was transfected into the 293 adenovirus packaging cell line. Virus was further propagated in 293 cells according to standard techniques. For all experiments, AdGFP (van Beusechem *et al*, 2000) was taken along as a negative control.

### Western blot analysis

Equivalent amounts of supernatant or cellular lysate from SW1398 cells transduced with Ad.C28-sCE2 were dissolved in sample buffer (Laemmli, 1970) with 2-mercaptoethanol and heated to 95°C for 5 min. Samples were electrophoresed through a denaturing 10% sodium dodecyl sulphate–polyacrylamide gel and protein bands were electroblotted onto a PVDF protein membrane (BioRad, Veenendaal, The Netherlands). Proteins were detected using anti-myc antibody 9E10 (Chan *et al*, 1987) and HRP-conjugated rabbit anti-mouse IgG (DakoCytomation, Heverlee, Belgium). Films were developed with enhanced chemoluminescence (Lumilight Plus, Roche, The Netherlands).

### Esterase activity assay

To evaluate the esterase activity of proteins expressed by SW1398 cells transduced with Ad.C28-sCE2, cellular lysates or supernatants were incubated with 200 *μ*l 100 mM Tris-HCl (pH=8.0) containing 1 mM *p*-nitrophenyl-acetate (pNpAc) (Sigma Aldrich, Zwijndrecht, The Netherlands), a substrate for CE. Conversion to pNp at room temperature was measured during 10 min using an ELISA plate reader (BioRad) at a wavelength of 415 nm.

### Immunohistochemistry

Spheroids were harvested at different time points after transduction (day 1, 4 or 5) in TissueTek (Sakura Finetek, Zoeterwoude, The Netherlands) and cryostat sections of 7–10 *μ*M were made and stored at −80°C. After drying, sections were fixed with 4% formaldehyde in PBS for 30 min, washed with PBS and treated with 0.2% Triton X-100 in PBS. After washing, the sections were incubated for 1 h with the anti-myc antibody 9E10. As a positive control, anti-EpCAM antibody 323A3 (kindly provided by Centocor, Leiden, The Netherlands) was taken along, and as negative controls, PBS/0.1% BSA and anti-glucuronidase (Haisma *et al*, 1995) were used. After incubation, sections were washed with PBS and incubated with rabbit-anti-mouse-HRP or goat-anti-rabbit-HRP (1 : 100 in PBS/0.1% BSA, both from DakoCytomation). After incubation for 1 h, sections were washed with PBS and stained with AEC (DakoCytomation) and sections were counterstained with haematoxylin.

### *In vitro* cytotoxicity assays

At 3 days after the formation of colon cancer spheroids, the spheroids were transduced with 1 × 10^7^ plaque-forming units (PFU) Ad.C28-sCE2 in 100 *μ*l culture medium. Control spheroids were transduced with AdGFP or cultured in medium. After 7 days, 100 *μ*l culture medium was added containing a range of CPT-11 (Aventis, Strasbourg, France). After a further 7 days, cell viability was determined by WST-1 (Roche Diagnostics) conversion at 37°C. Data are expressed as percentages compared to untransduced, untreated control spheroids.

## RESULTS

### Construction and characterisation of Ad.C28-sCE2

**Figure 1:**
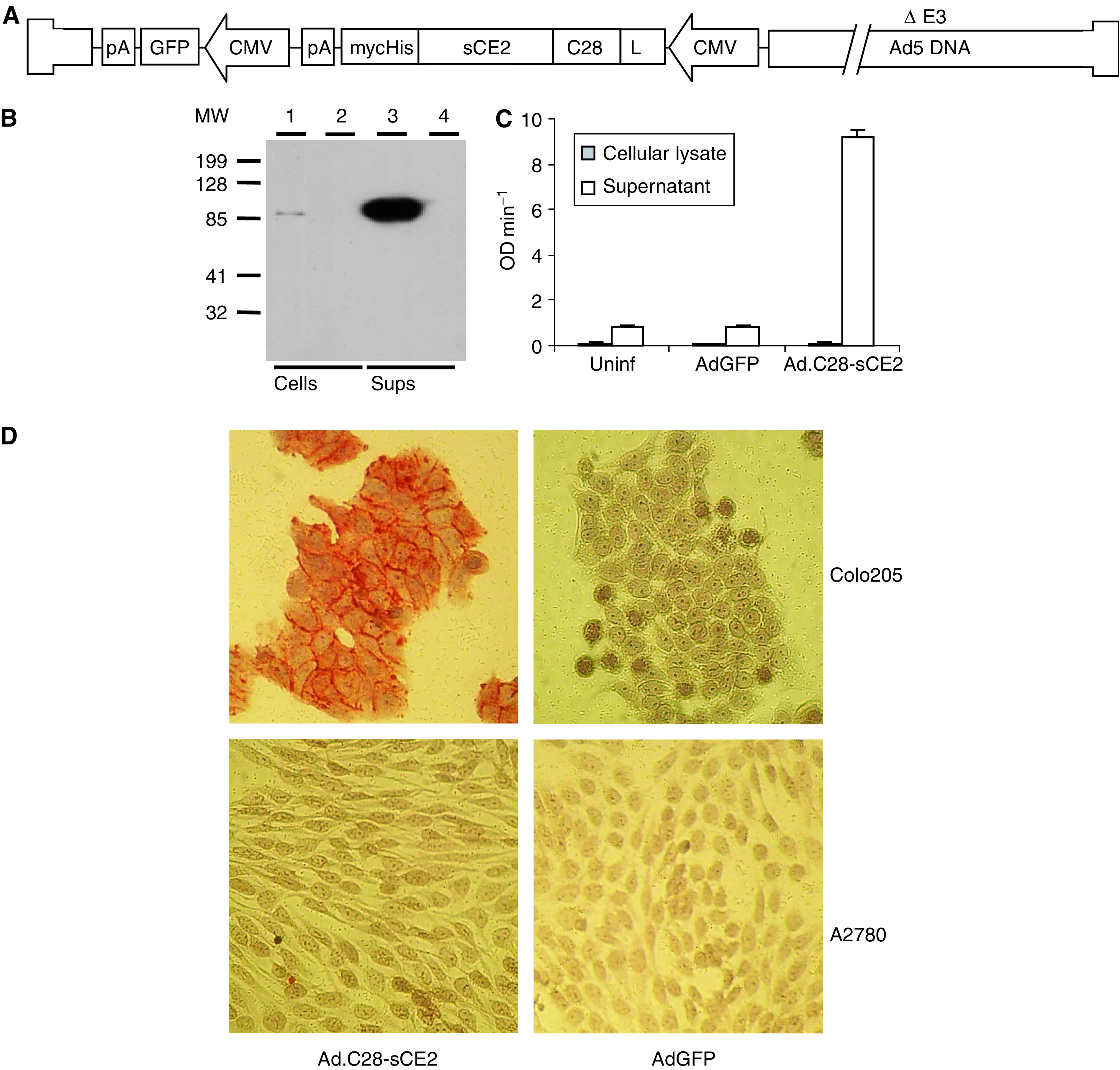
Schematic structure of the replication-deficient adenovirus Ad.C28-sCE2 and characterisation of Ad.C28-sCE2-transduced SW1398 cells by Western blot analysis, esterase activity assay and immunohistochemistry. (**A**) Schematic structure of the replication-deficient adenovirus Ad.C28-sCE2. The C28-sCE2 expression cassette includes the CMV promoter, an IgG*κ* leader sequence for secretion and a C-terminal myc- and His-tag for detection and purification. The adenovirus also contains the gene encoding GFP under the CMV promoter. (**B**) Western blot analysis of cellular lysates (lanes 1 and 2) and supernatants (lanes 3 and 4) of SW1398 cells transduced with Ad.C28-sCE2 (lanes 1 and 3) or AdGFP (lanes 2 and 4) at an MOI of 100. C28-sCE2 was detected using an antibody directed to the myc-tag. (**C**) CE activity in cellular lysates and supernatants of SW1398 cells transduced with Ad.C28-sCE2 or AdGFP at an MOI of 100. Cellular lysates or supernatants were incubated with 1 mM pNpAc and conversion was measured during 10 min. C28-sCE2 showed enzymatic activity and was efficiently secreted by transduced cells, since most of the activity was detected in the supernatant. (**D**) Binding of C28-sCE2 to the EpCAM-expressing cell line Colo205. Colo205 cells or the EpCAM-negative cell line A2780 were incubated with the supernatant of SW1398 cells transduced with Ad.C28-sCE2 or AdGFP at an MOI of 100. After washing, the cells were stained with anti-myc antibody to show binding of C28-sCE2. Only the EpCAM-expressing Colo205 cells incubated with supernatant of Ad.C28-sCE2-transduced SW1398 cells showed a positive membrane staining, indicating that the fusion protein had bound specifically to the Colo205 cells.

The open reading frame of C28-sCE2 with C-terminal mycHis-tag was inserted in place of the E1 region of an adenovirus vector next to a GFP expression cassette to create Ad.C28-sCE2 (Figure 1A).

SW1398 colon cancer cells were transduced with Ad.C28-sCE2 or control virus AdGFP at an MOI of 100 and after 6 days expression of sCE2 in supernatant and cellular lysate was analysed by Western blotting. Figure 1B shows that the majority of the 110 kDa C28-sCE2 protein was detected in the supernatant of Ad.C28-sCE2-transduced cells, confirming efficient secretion. Enzyme activity of C28-sCE2 was demonstrated by an esterase enzyme activity assay (Figure 1C). Binding of C28-sCE2 to EpCAM-expressing cells was shown by immunohistochemistry (Figure 1D). The EpCAM-positive cell line Colo205 and the EpCAM-negative ovarian cancer cell line A2780 were incubated with the supernatant of SW1398 cells transduced with Ad.C28-sCE2 or AdGFP. As can be seen in Figure 1D, C28-sCE2 specifically bound to the cellular membranes of EpCAM-expressing cells.

### Diffusion of C28-sCE2 in multicellular colon cancer tumour spheroids

**Figure 2:**
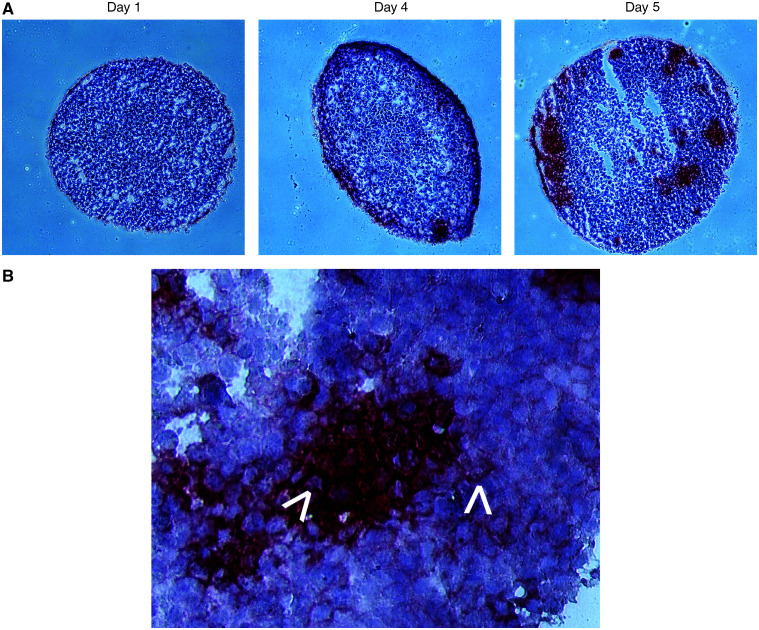
Immunohistochemistry on sections of Ad.C28-sCE2-transduced Colo205 spheroids. Colo205 spheroids were transduced with 1 × 10^7^ PFU Ad.C28-sCE2 and harvested at day 1, 4 and 5 after transduction. Sections of these spheroids were made and stained for myc to detect C28-sCE2. (**A**) At day 1 after Ad.C28-sCE2 transduction, no positive staining can be detected. At days 4 and 5, several spots along the rim of the spheroid are positively stained. (**B**) A higher magnification of the fusion protein staining at day 5 after transduction is shown. Cells with clear staining of membranes only (arrows) represent untransduced neighbouring cells with bound C28-sCE2.

Colo205 spheroids were transduced with Ad.C28-sCE2 and cryosections were made 1, 4 and 5 days later. Sections were stained with an anti-myc antibody to localise the C28-sCE2 fusion protein. Figure 2 illustrates that on day 1 after transduction only the outer rim of the spheroid stained slightly positive for C28-sCE2. Sections of spheroids harvested at later time points after transduction showed the presence of C28-sCE2 in deeper layers of the spheroid. A higher magnification of the anti-myc staining at day 5 after transduction (Figure 2B) suggests that C28-sCE2 had bound untransduced neighbouring cells since only the cellular membrane of these cells stained positive. Thus, C28-sCE2 penetrated into and accumulated in the tumour mass surrounding Ad.C28-sCE2-transduced cells.

### CPT-11 activation and antiproliferative effects in Ad.C28-sCE2-transduced cells

**Figure 3:**
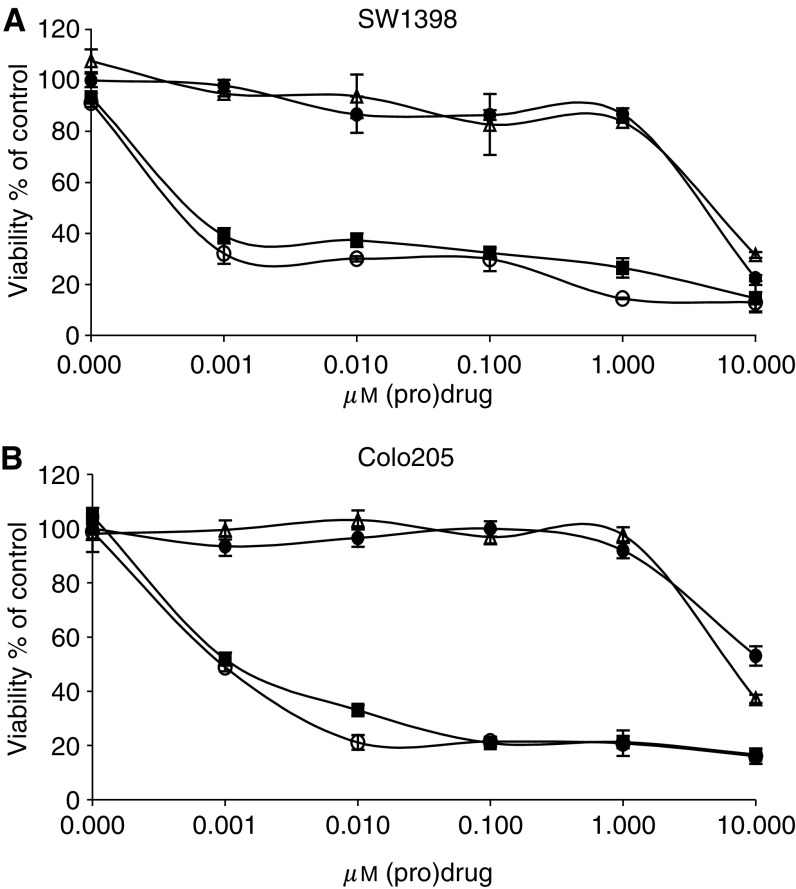
Cytotoxicity assay on SW1398 and Colo205 colon cancer spheroids. SW1398 (**A**) and Colo205 (**B**) spheroids were transduced with 1 × 10^7^ PFU AdGFP or Ad.C28-sCE2. At 7 days after infection, spheroids were subjected to a range of CPT-11 concentrations and cultured for a further 7 days. Cell viability of untransduced spheroids treated with CPT-11 (closed black circles) or SN-38 (open black circles), AdGFP-transduced spheroids treated with CPT-11 (open black triangles) and Ad.C28-sCE2-transduced spheroids treated with CPT-11 (closed black squares) were analysed by WST-1 conversion measurement.

Colon cancer spheroids transduced with Ad.C28-sCE2 or AdGFP were subjected to CPT-11 treatment for 7 days. Figure 3 demonstrated the viability of the spheroid as measured by WST-1 conversion. Ad.C28-sCE2-transduced Colo205 and SW1398 colon cancer spheroids were sensitised to CPT-11, since CPT-11 treatment to these spheroids was as toxic as treatment with its activate analogue SN-38.

## DISCUSSION

Targeting chemotherapy specifically to tumour cells with GDEPT is expected to increase the antitumour effect, while side effects are decreased. A limitation of adenoviral vector-mediated cancer gene therapy is the poor penetration ability of adenoviral vectors into a solid tumour mass. To improve the efficacy of adenoviral vector-mediated GDEPT approaches, secreted prodrug-converting enzymes have been studied (Weyel *et al*, 2000; Oosterhoff *et al*, 2003). However, secreted enzymes might leak away from the site of the tumour. Therefore, cell surface-tethered forms of prodrug-converting enzymes, such as *β*-glucuronidase or carboxypeptidase G2, were developed to prevent leakage of untargeted enzyme from the tumour, while prodrug activation is retained (Spooner *et al*, 2000; Heine *et al*, 2001). Another way to prevent diffusion of the enzyme from the tumour is secretion by transduced tumour cells of a fusion protein consisting of an scFv antibody and a prodrug-converting enzyme, which can subsequently bind to tumour cells (De Graaf *et al*, 2002; Oosterhoff *et al*, 2002). We hypothesised that the bystander effect achieved by such a secreted targeted prodrug-converting enzyme might be more pronounced than that achieved by a cell surface-tethered form, as the targeted form can diffuse and bind to neighbouring tumour cells. In this study, we investigated the utility of a replication-deficient adenoviral vector containing the cDNA encoding a secreted, EpCAM-targeted form of human liver CE2, Ad.C28-sCE2, to sensitise colon cancer tumours to CPT-11. We chose to study Ad.C28-sCE2 in a three-dimensional *in vitro* colon cancer spheroid model, because the three-dimensional structure of spheroids resembles *in vivo* tumours much closer than two-dimensional cell cultures. Furthermore, we wanted to visualise the bystander effect by determining secretion of C28-sCE2 and penetration of the fusion protein through a solid tumour mass, which can only be studied in a three-dimensional structure. Grill *et al* demonstrated that transduction of primary glioma spheroids with a replication-deficient vector resulted in the expression of the transgene in the outer rim of the spheroid only. This showed that spheroids are relevant structures to study lack of adenovirus penetration into solid tumour masses (Grill *et al*, 2002). In the colon cancer spheroid model used in this study, we were able to detect the C28-sCE2 fusion protein bound to untransduced cells several cellular layers away from transduced cells. This suggests that C28-sCE2 is capable of diffusing into a solid tumour mass.

From these results we hypothesised that optimal cytotoxicity from CPT-11 could be expected if the prodrug was administered at least a few days after Ad.C28-sCE2 transduction when C28-sCE2 has spread through the spheroid. Transduction of colon cancer spheroids with Ad.C28-sCE2 and treatment with CPT-11 after 7 days resulted in complete sensitisation of these spheroids to CPT-11. The toxicity to these spheroids was comparable to SN-38 treatment, indicating that CPT-11 is effectively converted into the toxic drug.

In order to compare a targeted prodrug-converting enzyme with a secreted prodrug-converting enzyme, it is necessary to perform *in vivo* experiments. However, the high endogenous plasma esterase activity in mice presents a challenge in using mouse models to evaluate tumour-specific conversion of CPT-11. In mice, more than 50% of the administered CPT-11 is converted to SN-38 by plasma esterases (Morton *et al*, 2000), whereas in human patients less than 5% of the prodrug is activated (Rivory *et al*, 1997). Hence, the analysis of CE-mediated activation of CPT-11 in normal mice does not accurately reflect what happens after the administration of the drug to humans. Previously, a strain of plasma esterase-deficient mice was described (Morton *et al*, 2000), in which CPT-11 metabolism is comparable to that observed in humans. Recently, these mice were crossbred with SCID mice (personal communication with Dr Phil Potter, St Jude Children's Research Hospital, Memphis, USA) and we are currently testing adenoviral vectors expressing EpCAM-targeted sCE2 or untargeted sCE2 in these esterase-deficient SCID mice bearing colon cancer xenografts.

In conclusion, we constructed a replication-deficient adenoviral vector containing a cDNA encoding a secreted, EpCAM-targeted form of human liver CE2 that was capable of converting the prodrug CPT-11 into its activated form, leading to enhanced toxicity of CPT-11 to colon cancer spheroids. Therefore, this adenoviral construct holds promise in GDEPT approaches for the treatment of patients with EpCAM-expressing colon cancer.
